# The Relationship between Zinc Status and Inflammatory Marker Levels in Rural Korean Adults Aged 40 and Older

**DOI:** 10.1371/journal.pone.0130016

**Published:** 2015-06-16

**Authors:** Sukyoung Jung, Mi Kyung Kim, Bo Youl Choi

**Affiliations:** 1 Department of Preventive Medicine, College of Medicine, Hanyang University, Seoul, South Korea; 2 Institute for Health and Society, Hanyang University, Seoul, South Korea; Oklahoma State University, UNITED STATES

## Abstract

**Background:**

Serum cytokines and C-reactive protein (CRP) are known as one of the major risk factors in atherosclerosis. The antioxidant and anti-inflammatory properties of zinc have been suggested, but few data are available on the relationship between zinc status and inflammatory markers in epidemiological studies.

**Objective:**

The present study aims to investigate the cross-sectional relationships of serum cytokines and CRP with dietary zinc intake and serum zinc levels in healthy men and women aged 40 and older in rural areas of South Korea.

**Materials and Methods:**

A group of 1,055 subjects (404 men, 651 women) was included in dietary zinc analysis while another group of 695 subjects (263 men, 432 women) was included in serum zinc analysis. Serum IL-6, TNF-α, and CRP were measured as inflammatory markers.

**Results:**

There was no significant inverse relationship between dietary zinc intake and inflammatory markers. We found a significant inverse relationship between serum zinc levels and all three inflammatory markers in women (*P* for trend = 0.0236 for IL-6; *P* for trend = 0.0017 for TNF-α; *P* for trend = 0.0301 for CRP) and between serum zinc levels and a single inflammatory marker (IL-6) in men (*P* for trend = 0.0191), although all R2 values by regression were less than 10%.

**Conclusion:**

In conclusion, serum zinc levels may be inversely related to inflammatory markers (IL-6, TNF-α, and CRP), particularly in women.

## Introduction

Cytokines are a large group of molecules involved in cellular signaling during immune responses [1] and thought to be involved in every step of atherosclerosis [2,3], an inflammatory process as well as an independent risk factor for cardiovascular disease (CVD) [2]. Cytokines are classified into several families [4] and among them, interleukin-1 (IL-1), interleukin-6 (IL-6), and tumor necrosis factor alpha (TNF-α) being recognized as the primary pro-inflammatory cytokines that promote inflammation [5]. In the liver, IL-6 predominantly stimulates the secretion of C-reactive protein (CRP), an acute phase protein [6] that acts as a sensitive systemic marker indicating inflammation and tissue damage [7] and is also related to CVD risk via its involvement in inflammatory mechanisms [8].

Although zinc deficiency is not considered a public health issue in developed countries, zinc remains an important nutrient due to its antioxidant and anti-inflammatory properties [9]. Despite these properties of the zinc, few data are available on the relationship between zinc status and inflammatory markers in epidemiological studies and the results of the few studies conducted on this topic have been inconsistent [9,10]. Previous studies reported a positive cross-sectional relationship between dietary zinc intake and CRP levels in healthy American subjects aged 45–84 [9] and an inverse cross-sectional relationship between serum zinc levels and CRP levels in very elderly Brazilian subjects [10].

However, two Korean studies have indicated low dietary zinc intake [11] and low zinc bioavailability using a phytate:zinc molar ratio [12] were related to subclinical atherosclerosis and these studies suggested a potential protective effect of dietary zinc on atherosclerosis. Understanding the association between zinc status and the inflammation process as the early stages of atherosclerosis may provide further information on how to protect against atherosclerotic progression as well as CVD.

The aim of the present study is to investigate the relationship of IL-6, TNF-α, and CRP with dietary zinc intake and serum zinc levels in healthy men and women aged 40 years and older living in rural areas of South Korea.

## Materials and Methods

### Study population

This study population was a part of the ongoing The **Ko**rean **G**enome **E**pidemiology **S**tudy (KoGES), initiated to identify risk factors for cardiovascular disease. Subjects in the present study included 1,703 adults aged 40 years and over living in Yangpyeong County located 45 km east of Seoul, the capital of South Korea. Subjects were recruited to investigate the subclinical atherosclerosis risk factors between January 2005 and December 2009. The majority of the subjects were self-reported farmers and housewives. To identify the relationship between zinc status and inflammatory markers, subjects who did not provide sufficient specimen to detect serum inflammatory markers (n = 109), subjects with a history of heart disease, stroke, and/or cancer (physician-diagnosed) (n = 122), and subjects taking medication for hypertension, diabetes mellitus, and/or dyslipidemia (n = 399) were excluded. Additionally, we excluded subjects who reported implausible dietary intakes (<500 or >4000 kcal/d) (n = 4) as well as those who did not have data on alcohol intake (n = 4), blood pressure (n = 2), anthropometric measurements (height, weight, waist circumference) (n = 2), education level (n = 2), and/or exercise habits (n = 2). Additionally, extreme outliers for serum inflammatory markers (n = 2) were removed. Finally, 1,055 subjects (404 men, 651 women) were included in the final analysis of dietary zinc intake and 695 subjects (263 men, 432 women) who were available serum zinc data were included in the final analysis of serum zinc levels. This study was approved by the Institutional Review Board of Hanyang University and was conducted in accordance with the Declaration of Helsinki. All subjects provided written informed consent to participate in this study.

### General characteristics and anthropometric measurements

To determine general characteristics, including information on demographics, education, smoking status, alcohol consumption, exercise habits, medical history, and menstrual history, a structured questionnaire was administered by trained interviewers. Educational level, which has been reported as a stronger indicator among other indicators reflecting socioeconomic status, was used as an index of socioeconomic position [13]. Higher education was defined as more than 12 years of schooling, while regular exercise was defined as ≥ 3 times per week and ≥ 30 min per session. Smoking status was classified as either current smoker or non-smoker (including past smokers). Study subjects were asked their average frequency of alcohol consumption and the average amount of alcohol consumed to estimate daily alcohol consumption. Total daily alcohol consumption was calculated based on the total volume of all alcoholic beverages consumed, as expressed in grams of alcohol per day (g/d).

Height was measured with a standard height scale to the nearest 0.1 cm and weight was measured with a metric weight scale to the nearest 0.01 kg with subjects in light clothing without shoes. Body mass index was calculated as weight in kg divided by the square of height in meters (kg/m^2^). Waist circumference was measured halfway between the lowest rib margin and the iliac crest. Blood pressure (BP) was measured in a seated position via auscultation using a standard sphygmomanometer and cuff on the right arm. Two consecutive BP measurements were taken after each subject had been sitting for at least 5 minutes. Systolic blood pressure (SBP) and diastolic blood pressure (DBP) were measured with a standard mercury sphygmomanometer using the first and fifth Korotkoff sounds to the nearest 2 mmHg. If the two systolic or diastolic blood pressure readings were more than 5 mmHg apart, an additional measurement was performed and the mean value of the last two measurements was used in subsequent analysis.

### Biochemical analyses

Blood samples were collected in the morning after at least eight hours of fasting and all biochemical markers were analyzed on the same day. Serum cytokines (IL-1β, IL-6, and TNF-α) were measured using the multiplex method (Luminex 200, Luminex Corp, USA) and ranged from 0.14–400 pg/mL for IL-1β, 0.11–400 pg/mL for IL-6, and 0.16–400 pg/mL for TNF-α. Intra-assay precision is generated from the mean of the % coefficient of variation (V)'s from sixteen reportable results across two different concentration of cytokines in a single assay. Inter-assay precision is generated from the mean of the % CV's from four reportable results across two different concentrations of cytokines across six different experiments. Intra- and inter-assay CVs were 11.9 vs 10.3% for IL-6; 10.6 vs 9.8% for TNF-α, respectively. CRP was assayed by Turbidimetric immunoassay for high sensitivity CRP using ADVIA 1650 auto analyzer (Siemens medical sol.,USA) with a range from 0.2-400mg/L. Serum zinc levels were measured via inductively coupled plasma mass spectroscopy (ICP-MS; ICP-Mass, Bruker 820-MS, Australia) with a range from 66.0–110.0 μg/dL.

### Dietary intake assessment

Food and nutrient intakes were estimated using a food frequency questionnaire (FFQ) with 106 food items. Subjects were asked to identify how frequently they consumed 106 food items during the past year as well as the average amount they consumed. On the FFQ, nine frequency categories ranging from “never or rarely” to “3 times/d” were used to determine frequency of consumption and three serving sizes were listed to determine the amount of consumption. For food items with limited seasonal availability, subjects were asked to indicate whether they ate them for 3, 6, 9, or 12 months of the year. The validity and reproducibility of the FFQ has been examined in detail elsewhere [14]. Nutrient intake including zinc was calculated using a weighted frequency per day and serving size per unit for each food item. This study used nutrient database from the seventh edition of the Korean Food Composition Table [15], while the nutrient database [16] from the sixth edition of the Korean Food Composition Table was used in our previous study [12]. Calculations of the mean daily zinc intake values using both versions of the nutrient database resulted in some slight differences (7.4 and 9.6 mg/d in men; 6.2 and 8.0 mg/d in women). However, high correlation (r = 0.9456, *P*-value < 0.0001 in men; r = 0.9000, *P*-value < 0.0001 in women) and similar patterns of the main results were consistently observed in both sets of data (data not shown).

Dietary phytate intake was estimated using a phytate database of commonly consumed foods in Korea [17] and the United States [18]. For foods with no phytate content information [17,18], phytate values for different forms of the same food or similar foods were substituted. The details of this substitution method were reported elsewhere [12].

### Statistical analysis

Extreme outlier cytokines include those with values ten times higher than the value of the 99^th^ percentile [19]. One extreme outlier for IL-1β was greater than 83.1 pg/mL (p99 = 8.31) in men while one outlier for TNF-α was greater than 200 pg/mL (p99 = 20.0) in women. These values were excluded from the final analysis. As recommended in previous studies [20,21], all IL-1β, IL-6, and TNF-α values measured below the detection level (0.2 pg/mL) were substituted with a value (0.1) equivalent to half of the lower detection limit [19]. For IL-1β, however, 87% of the subjects exhibited values lower than the detection level, so we excluded IL-1β from the present study. IL-6, TNF-α, and CRP were log transformed for analysis because of right skewness.

Nutrient intakes were adjusted for total energy intake using a residual method, which is based on the simple relationship between nutrient intake and total energy intake separately in men and women [22]. Subjects were categorized into tertiles based on daily zinc intake and serum zinc levels.

The general characteristics of the subjects are described as the average and standard deviation for continuous variables and in terms of prevalence for categorical variables. To assess potential confounders that could affect the relationship between zinc status and inflammatory markers, age-adjusted averages or prevalence were obtained by the zinc status groups using the general linear model (GLM) and Cochran Mantel Haenszel analysis. Tukey's post-hoc comparison test was used to identify group differences at *P*-value < 0.05. Trend tests were conducted by treating the median value of each group as a continuous variable in the age-adjusted model. Variables showing significant linear trends across dietary zinc intake and serum zinc level groups were included in analysis as potential confounders. Two different models were applied to the analysis of dietary zinc intake and inflammatory markers. In the first model, age was the only variable adjusted. With the exception of dietary variables, variables that demonstrated significant linear trends across tertiles of dietary zinc intake were adjusted in the second model (age and alcohol intake for men; age, alcohol intake, waist circumference, higher education, and smoking status for women). Because there were no significant linear trends across tertiles of serum zinc level, only the age-adjusted model was applied in serum zinc levels analysis.

To compare the means of inflammatory markers (IL-6, TNF-α, and CRP) according to dietary zinc intake and serum zinc level groups, the GLM was used and Tukey’s post-hoc comparison test was used to identify group differences at *P*-value < 0.05. Regression coefficients and R^2^ were also presented using multiple linear regression analysis. Trend tests were conducted by treating the median value of each group as a continuous variable in multivariable-adjusted models.

SAS software (Version 9.3, SAS Institute Inc.; Cary, NC, USA) was used for all statistical analyses. *P*-values < 0.05 were considered significant.

## Results

General characteristics of the study subjects are shown in Table 1. The mean ages of the men and women were 60.3 and 57.3 y, respectively. The proportions of subjects with higher education and who were current smokers and current drinkers were higher among men than among women. Men had a higher mean waist circumference than women. Mean IL-6, TNF-α, and CRP values were higher among men than women. The mean daily zinc intake for men and women was 9.6 and 8.0 mg, respectively. These characteristics were similar to those of subjects in serum zinc analysis and the average of serum zinc was 105.2 μg/dL (SD, 16.6) for men and 104.7 μg/dL (SD, 14.9) for women (data not shown).

**Table 1:** General characteristics of the study subjects^1^.

Characteristics	Men	Women
n	404	651
Age (y)	60.3 ± 9.5	57.3 ± 9.8
Higher education (n, %)^2^	135 (33.4)	160 (24.6)
Regular exercise (n, %)^3^	78 (19.3)	159 (24.4)
Current smoker (n, %)	124 (30.7)	10 (1.5)
Alcohol consumption		
Current drinker (n, %)	272 (67.3)	223 (34.3)
Alcohol intake (g/d)	28.5 ± 49.5	2.3 ± 6.7
Body mass index (kg/m^2^)	23.9 ± 2.9	24.7 ± 3.1
Waist circumference (cm)	86.1 ± 8.0	84.0 ± 8.8
Menopausal women (n, %)	-	475 (73.2)
Seated blood pressure (mmHg)		
Systolic	122.6 ± 14.0	118.8 ± 16.9
Diastolic	80.5 ± 9.7	77.7 ± 10.1
IL-6 (pg/mL)^4^	2.60 ± 2.28	2.38 ± 2.36
TNF-α (pg/mL)^4^	7.06 ± 1.63	6.42 ± 1.59
CRP (mg/L)^4^	2.19 ± 1.89	2.00 ± 1.69
**Dietary intake**		
Energy (kcal/d)	1804.2 ± 532.2	1520.7 ± 432.6
Protein (g/d)	57.2 ± 9.7	47.9 ± 8.1
Fat (g/d)	23.4 ± 8.9	17.2 ± 7.5
Carbohydrate (g/d)	346.7 ± 36.4	300.9 ± 31.7
β-carotene (μg/d)	2651.9 ± 1431.2	2444.8 ± 1412.0
Vitamin E (mg/d)	7.3 ± 1.7	6.0 ± 1.6
Vitamin C (mg/d)	85.3 ± 40.6	85.9 ± 43.1
Folate (μg/d)	452.2 ± 127.8	414.6 ± 123.4
Zinc (mg/d)	9.6 ± 2.0	8.0 ± 1.3
Phytate (mg/d)	689.8 ± 181.4	614.9 ± 143.7

IL-6, interleukin 6; TNF-α, tumor necrosis factor-alpha; CRP, C-reactive protein;

^1^ Values are expressed as the mean ± SD or number (%)

^2^≥ High school graduates (12 years of education)

^3^ ≥3 times/week and ≥30 min/session

^4^ Mean values of IL-6, TNF-a, and CRP are back-transformed values.

**Fig 1:**
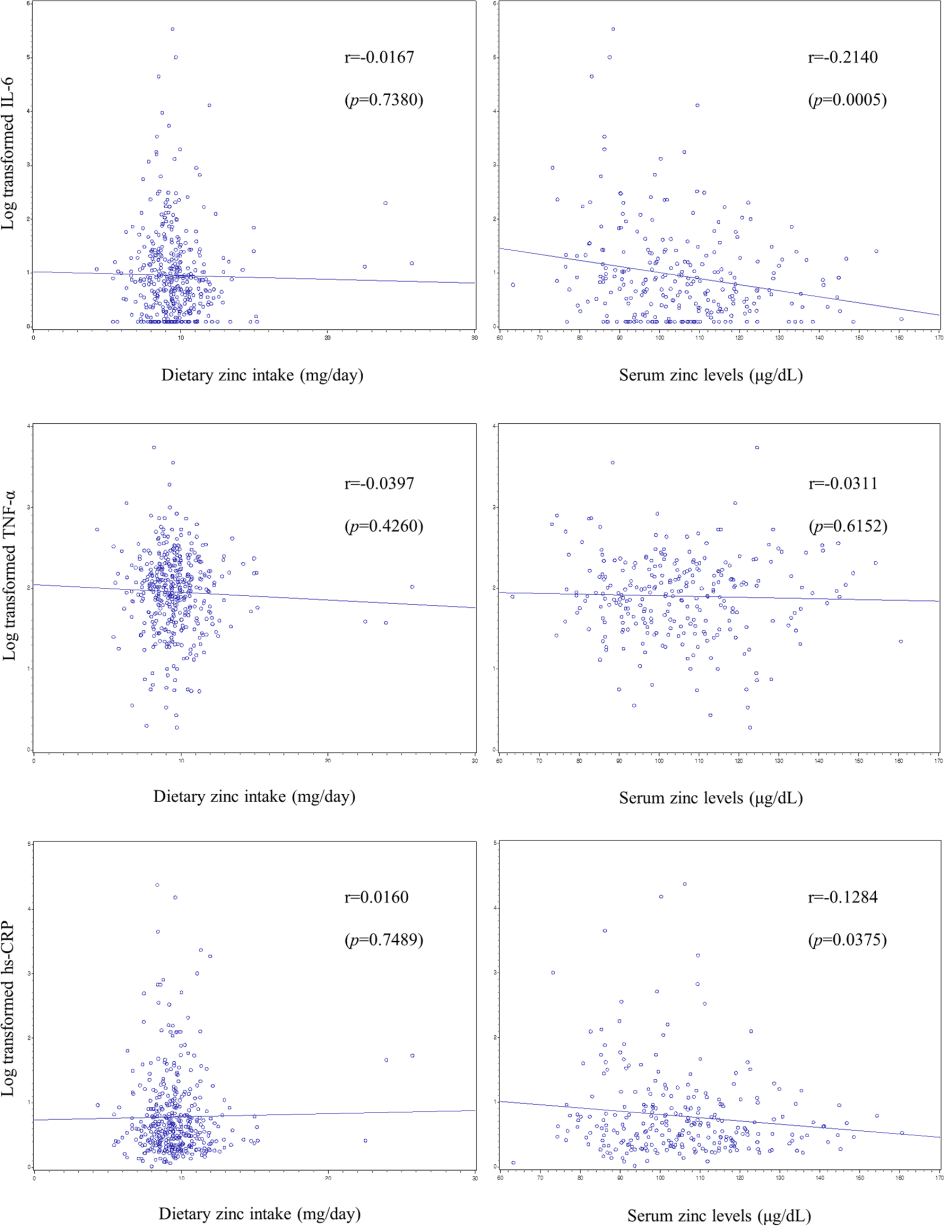
Scatter plots of dietary zinc intake and serum zinc levels with serum cytokines and C-reactive protein in men.

**Fig 2:**
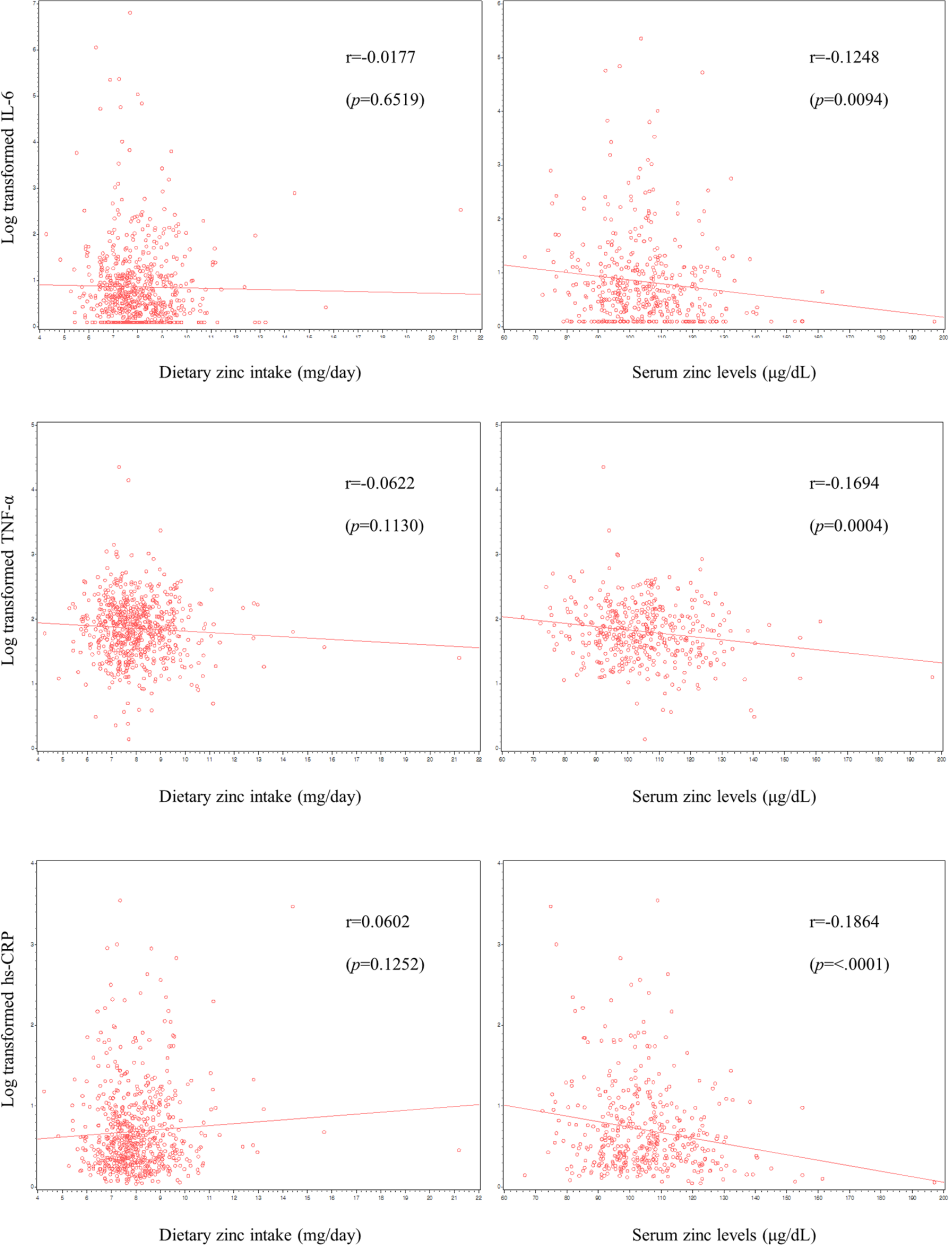
Scatter plots of dietary zinc intake and serum zinc levels with serum cytokines and C-reactive protein in women.

Figs 1 and 2 demonstrate correlations of dietary and serum zinc with inflammatory markers. Serum zinc levels exhibited significant negative correlation with IL-6 and CRP in men, with IL-6, TNF-α, and CRP in women.

Potential confounders for dietary zinc intake and serum zinc level groups are shown in Table 2. Higher education, regular exercise, waist circumference, and phytate intake in men and regular exercise, daily alcohol consumption, waist circumference, and phytate intake in women showed significant linear trends across dietary zinc intake tertile groups. None of the variables demonstrated a significant linear trend across serum zinc level tertile groups.

**Table 2:** Age-adjusted characteristics of the study subjects according to dietary zinc intake and serum zinc levels^1^.

	Tertiles of dietary zinc intake (mg/d)					Tertiles of serum zinc levels (μg/dL)			
Characteristics	T1	T2	T3	*P* for linear trend^2^	Characteristics	T1	T2	T3	*P* for linear trend^2^
***Men (n = 404)***					***Men (n = 263)***				
n	134	135	135		n	87	88	88	
Median of zinc intake	8.10	9.42	10.92		Median of serum zinc level	88.9	103.9	119.9	
Range of zinc intake	4.30–8.81	8.83–9.94	9.94–25.74		Range of serum zinc level	63.3–96.8	97.1–110.2	110.7–160.6	
Age (y)	60.9 ± 0.8	60.5 ± 0.8	59.5 ± 0.8	0.2090	Age (y)	64.1 ± 1.0^a^	60.1 ± 1.0^b^	59.8 ± 1.0^b^	0.0025
Higher education (%)^3^	26.4^a^	30.7^ab^	43.0^b^	0.0018	Higher education (%)^3^	37.6	29.4	31.1	0.3563
Regular exercise (%)^4^	11.4^a^	19.3^ab^	27.1^b^	0.0011	Regular exercise (%)^4^	27.4	16.1	19.3	0.2168
Current smoker (%)	35.5	31.3	25.4	0.0711	Current smoker (%)	30.7	24.1	29.6	0.9126
Current drinker (%)	63.1	66.1	72.8	0.0846	Current drinker (%)	70.3	67.6	61.7	0.2339
Alcohol intake (g/d)	28.0 ± 4.3	23.1 ± 4.2	34.8 ± 4.2	0.2337	Alcohol intake (g/d)	37.9 ± 5.4	23.8 ± 5.3	23.9 ± 5.3	0.0756
Body mass index (kg/m^2^)	23.5 ± 0.2	24.0 ± 0.2	24.2 ± 0.2	0.0387	Body mass index (kg/m^2^)	23.8 ± 0.3	24.3 ± 0.3	23.8 ± 0.3	0.9387
Waist circumference (cm)	84.5 ± 0.7^a^	86.2 ± 0.7^ab^	87.7 ± 0.7^b^	0.0009	Waist circumference (cm)	85.2 ± 0.9	88.0 ± 0.8	87.5 ± 0.9	0.0716
Dietary phytate (mg/d)	601.7 ± 14.8^a^	720.0 ± 14.7^b^	747.1 ± 14.7^b^	<.0001	Dietary phytate (mg/d)	675.1 ± 20.4	722.1 ± 20.1	728.1 ± 20.1	0.0731
***Women (n = 651)***					***Women (n = 432)***				
n	217	217	217		n	142	147	143	
Median of zinc intake	6.96	7.83	9.06		Median of serum zinc level	91.8	103.6	118.3	
Range of zinc intake	4.27–7.39	7.40–8.35	8.35–21.19		Range of serum zinc level	66.6–97.3	97.4–108.6	108.7–197.1	
Age (y)	59.2 ± 0.7^a^	57.2 ± 0.7^ab^	55.4 ± 0.7^b^	<.0001	Age (y)	58.2 ± 0.8^ab^	59.5 ± 0.8^a^	56.2 ± 0.8^b^	0.0470
Menopausal status (%)	72.3	74.1	73.2	0.8049	Menopausal status (%)	74.7	75.0	81.3	0.0651
Higher education (%)^3^	23.2	24.2	26.3	0.4021	Higher education (%)^3^	21.6^ab^	27.5^a^	14.5^b^	0.0780
Regular exercise (%)^4^	19.2^a^	22.5^ab^	31.6^b^	0.0023	Regular exercise (%)^4^	24.1	25.6	24.6	0.9373
Current smoker (%)	2.7	0.0	1.9	0.6401	Current smoker (%)	0.7	0.6	0.8	0.9327
Current drinker (%)	29.9^a^	30.8^a^	42.0^b^	0.0055	Current drinker (%)	34.0	35.7	31.7	0.6607
Alcohol intake (g/d)	2.0 ± 0.5^a^	1.5 ± 0.4^a^	3.6 ± 0.5^b^	0.0067	Alcohol intake (g/d)	2.7 ± 0.6	2.3 ± 0.6	2.0 ± 0.6	0.4104
Body mass index (kg/m^2^)	24.4 ± 0.2	24.6 ± 0.2	25.0 ± 0.2	0.0743	Body mass index (kg/m^2^)	24.8 ± 0.3	24.7 ± 0.3	24.9 ± 0.3	0.8805
Waist circumference (cm)	82.8 ± 0.6^a^	84.0 ± 0.6^ab^	85.1 ± 0.6^b^	0.0069	Waist circumference (cm)	84.4 ± 0.7	85.6 ± 0.7	86.1 ± 0.7	0.0759
Dietary phytate (mg/d)	562.4 ± 9.5^a^	637.1 ± 9.4^b^	644.1 ± 9.5^b^	<.0001	Dietary phytate (mg/d)	602.7 ± 12.3	634.1 ± 12.1	628.4 ± 12.3	0.1608

^1^ All results except each median value and age were adjusted for age, and all nutrient intakes are total energy-adjusted values. Values are expressed as the mean ± SE or percentage (%). Mean values with different superscripts (^a^, ^b^, ^c^) within a row were significantly different among the three groups by Tukey’s multiple comparison test.

^2^ *P* for linear trend was determined by the general linear model for continuous variables and by the Cochran-Mantel-Haenszel test for categorical variables.

^3^≥ High school graduates (12 years of education)

^4^ ≥3 times/wk and ≥ 30 min/session

Relationships between dietary zinc intake and inflammatory markers are shown in Table 3. An age-adjusted analysis revealed that dietary zinc intake had a significantly inverse relationship with serum IL-6 in men (*P* for trend = 0.0477), but not in women. This significant linear trend diminished in multivariable models. No significant relationship between dietary zinc intake and serum TNF-α was found in both men and women. Multiple linear regression analysis indicated that a 1-mg increase in the daily consumption of zinc was related to a mean increase of 0.0335 pg/mL in CRP after adjustment for age in women (*P*-value = 0.0270), but not in men. In the multivariable analysis, no significant results were obtained for both men and women (Table 3).

**Table 3:** Serum cytokines and C-reactive protein of the study subjects according to daily zinc intake^1^.

	Tertiles of dietary zinc intake (mg/d)						
	T1	T2	T3	*P* for linear trend^2^	β	R^2^	*P*-value^3^
***Men (n = 404)***							
n	134	135	135				
Median of zinc intake (mg/d)	8.10	9.42	10.92				
Range of zinc intake (mg/d)	4.30–8.81	8.83–9.94	9.94–25.74				
***Interleukin-6 (pg/mL)***							
Age-adjusted IL-6	2.80 ± 1.07	2.72 ± 1.07	2.32 ± 1.07	0.0477	-0.0044	5.24	0.8240
Multivariable-adjusted IL-6^4^	2.86 ± 1.08	2.92 ± 1.08	2.46 ± 1.08	0.1350	0.0068	7.23	0.7462
***Tumor necrosis factor-alpha (pg/mL)***							
Age-adjusted TNF-α	7.32 ± 1.04	7.10 ± 1.04	6.82 ± 1.04	0.2696	-0.0097	0.25	0.4177
Multivariable-adjusted TNF-α^4^	7.77 ± 1.05	7.32 ± 1.05	6.89 ± 1.05	0.0740	-0.0176	1.75	0.1644
***C-reactive protein (mg/dL)***							
Age-adjusted CRP	2.25 ± 1.05	2.18 ± 1.05	2.12 ± 1.05	0.4190	0.0060	1.58	0.6990
Multivariable-adjusted CRP^4^	2.16 ± 1.07	2.12 ± 1.06	2.08 ± 1.06	0.5585	0.0091	2.77	0.5833
***Women (n = 651)***							
n	217	217	217				
Median of zinc intake (mg/d)	6.96	7.83	9.06				
Range of zinc intake (mg/d)	4.27–7.39	7.40–8.35	8.35–21.19				
***Interleukin-6 (pg/mL)***							
Age-adjusted IL-6	2.59 ± 1.06	2.20 ± 1.06	2.36 ± 1.06	0.3245	-0.0004	1.92	0.9885
Multivariable-adjusted IL-6^4^	2.48 ± 1.06	2.16 ± 1.06	2.32 ± 1.06	0.5933	0.01082	3.20	0.6721
***Tumor necrosis factor-alpha (pg/mL)***							
Age-adjusted TNF-α	6.62 ± 1.03	6.23 ± 1.03	6.36 ± 1.03	0.4303	-0.0177	1.10	0.1900
Multivariable-adjusted TNF-α^4^	6.62 ± 1.03	6.30 ± 1.03	6.42 ± 1.03	0.6776	-0.0142	1.85	0.3048
***C-reactive protein (mg/dL)***							
Age-adjusted CRP	2.01 ± 1.04^ab^	1.84 ± 1.04^a^	2.16 ± 1.04^b^	0.1102	0.0335	4.57	0.0270
Multivariable-adjusted CRP^4^	2.05 ± 1.04^ab^	1.84 ± 1.04^a^	2.10 ± 1.04^b^	0.3892	0.0265	11.15	0.0782

^1^ Values are expressed as the mean ± SE. Mean values of IL-6, TNF-a, and CRP are back-transformed values. Mean values with different superscripts (^a^, ^b^, ^c^) within a row are significantly different among the three groups by Tukey’s multiple comparison test.

^2^ *P* for linear trend was determined by treating the median value of each group as a continuous variable using the general linear model (GLM).

^3^ *P* values, regression coefficients, and R^2^ were obtained by multiple linear regression analysis.

^4^ The multivariable model for dietary zinc included age (years), education (≥12 years), regular exercise (yes/no), waist circumference (cm), phytate intake (mg/d) among men and age (years), regular exercise (yes/no), daily alcohol consumption (g/d), waist circumference (cm) and phytate intake (mg/d) among women.

Table 4 presents the relationship between serum zinc levels and inflammatory markers. Serum zinc levels in men subjects exhibited a significant inverse relationship with IL-6 (*P* for trend = 0.0191, β = -0.0901 (*P*-value = 0.0048)), but no significant relationships were found between serum zinc levels and TNF-α or CRP. In women, serum zinc levels had a significant inverse relationship with IL-6, TNF-α, and CRP levels in both analyses (*P* for trend = 0.0236, β = -0.0571, R^2^ = 3.79 (*P*-value = 0.0321) for IL-6; *P* for trend = 0.0017, β = -0.0464, R^2^ = 4.10 (*P*-value = 0.0015) for TNF-α; *P* for trend = 0.0301, β = -0.0560, R^2^ = 8.68 (*P*-value = 0.0011) for CRP).

**Table 4:** Serum cytokines and C-reactive protein of the study subjects according to serum zinc levels^1^.

	Tertiles of serum zinc level (μg/dL)						
	T1	T2	T3	*P* for linear trend^2^	β	R^2^	*P*-value^3^
***Men (n = 263)***							
n	87	88	88				
Median of serum zinc level (μg/dL)	88.9	103.9	119.9				
Range of serum zinc level (μg/dL)	63.3–96.8	97.1–110.2	110.7–160.6				
***Interleukin-6 (pg/mL)***							
Age-adjusted IL-6	3.06 ± 1.09^a^	2.53 ± 1.09^ab^	2.25 ± 1.09^b^	0.0191	-0.0901	9.83	0.0048
***Tumor necrosis factor-alpha (pg/mL)***							
Age-adjusted TNF-α	6.69 ± 1.06	6.89 ± 1.05	6.55 ± 1.05	0.8235	-0.0075	0.22	0.6980
***C-reactive protein (mg/dL)***							
Age-adjusted CRP	2.23 ± 1.07^ab^	2.48 ± 1.07^a^	1.92 ± 1.07^b^	0.1069	-0.0468	1.90	0.0585
***Women (n = 432)***							
n	142	147	143				
Median of serum zinc level (μg/dL)	91.8	103.6	118.3				
Range of serum zinc level (μg/dL)	66.6–97.3	97.4–108.6	108.7–197.1				
***Interleukin-6 (pg/mL)***							
Age-adjusted IL-6	2.46 ± 1.07^ab^	2.51 ± 1.07^a^	1.99 ± 1.07^b^	0.0236	-0.0571	3.79	0.0321
***Tumor necrosis factor-alpha (pg/mL)***							
Age-adjusted TNF-α	6.69 ± 1.04^a^	6.11 ± 1.04^ab^	5.64 ± 1.04^b^	0.0017	-0.0464	4.10	0.0015
***C-reactive protein (mg/dL)***							
Age-adjusted CRP	2.16 ± 1.04	2.05 ± 1.04	1.90 ± 1.04	0.0301	-0.0560	8.68	0.0011

^1^ Values are expressed as the mean ± SE. Mean values of IL-6, TNF-a, and CRP are back-transformed values. Mean values with different superscripts (^a^, ^b^, ^c^) within a row are significantly different among the three groups by Tukey’s multiple comparison test.

^2^ *P* for linear trend was determined by treating the median value of each group as a continuous variable using the general linear model (GLM).

^3^ *P* values, regression coefficients, and R^2^ were obtained by multiple linear regression analysis.

## Discussion

In this cross-sectional study of zinc status and inflammatory markers, we found a significant inverse relationship of serum zinc levels with all three inflammatory markers (IL-6, TNF-α, and CRP) in women and with only IL-6 in men. No significant inverse relationships were found between dietary zinc intake and inflammatory markers.

Although zinc consumption was calculated from FFQ and consequently was not recognized as absolute intake, average zinc intake (9.6 mg/d for men and 8.0 mg/d for women) in the present study was higher than the Korean estimated average requirements (EAR) for dietary zinc [23], and that of lower than European sample of elderly people (Austria, Denmark, Germany, Italy, and UK) [24] and US population aged over 40 years using NHANES 2011–2012 [25]. Zinc status can be assessed by biochemical, and functional indicators as well as dietary intake [26]. Of these indicators, biochemical indicators had a characteristic of an objective and quantitative when assessing the zinc status [26]. Biochemical indicators include concentrations of plasma zinc, urinary zinc excretion, erythrocyte zinc, mononuclear cell zinc, polymorphonuclear cell zinc, platelet zinc, hair zinc, plasma alkaline phosphatase activity, and metallothionein [27]. Serum zinc levels have been identified as an optimal marker to assess the zinc status at a population level [28,29]. Though no accurate marker of zinc status of an individual currently exists, serum zinc levels are considered as the easiest and most informative marker to use in assessing zinc status [30], particularly at the individual level in large population studies [31]. Serum zinc level (μg/dL) of the present study subjects (89.9–119.9 for men and 91.8–118.3 for women) was lower than that of Korean population aged over 20 years using KNHANES 2010 data (128.8–180.0 for men and 118.2–166.9 for women) [32] and similar to very elderly Brazilian individuals (81.0–120.0 for total subjects) [10].

In the present study, there was no correlation between dietary zinc intake and serum zinc levels (r = -0.0641, *P*-value = 0.3014 in men; r = 0.0037, *P*-value = 0.9397 in women). Similarly, this correlation was also found among Japanese adult populations [33] and New Zealand women 18–40 years of age who did not use oral contraceptives [34]. However, two other studies showed a positive correlation in Europeans [30] and the dose-response relationship between zinc supplements and blood zinc levels in meta-analysis [35]. In a subsample of very elderly Brazilian individuals, average zinc intake did not vary significantly according to serum zinc level tertiles [10]. Taken together [10,30,33–35], these contrasting results indicate that the true nature of the relationship between dietary zinc intake and serum or plasma zinc level remains unclear.

By the way, in the present study, inflammatory markers were not associated with dietary zinc intake, but showed significant inverse associations with serum zinc levels. The discrepancy between dietary zinc intake and serum zinc status may be partially explained by properties as follows; first, serum zinc levels may reflect recent dietary zinc intake better than long-term intake as suggested in a previous *in vivo* study [36], although chronic low zinc intake may cause the zinc deficiency in which case serum zinc levels can be indicative of a population’s risk for zinc deficiency [28]. Second, inflammatory markers (pro-inflammatory cytokines and CRP) in the present study may be responsible for acute inflammation [3,6]. The low correlation between dietary zinc intake and serum zinc status may also attribute to those properties.

There were previous studies reporting the inverse relationships of long term dietary zinc intake with the incidence of [37] and mortality related to CVD [38]. We yielded meaningful results indicating an inverse association between dietary zinc intake using FFQ and subclinical atherosclerosis index [11,12] However, limited prior study of the epidemiological relationship between dietary zinc intake or serum zinc levels and inflammatory markers has been conducted [9,10]. Among inflammatory markers, CRP has been examined its relation to dietary zinc intake among US healthy populations reporting a significantly positive relation to CRP levels [9] and to serum zinc status among elderly Brazilian populations which showed an inverse relationship [10]. To the best of our knowledge, and the present study was the first report of the relationship between serum zinc levels and inflammatory markers such as IL-6 and TNF-α.

In the present study, we found a significant relation between serum zinc status and inflammatory markers, although serum zinc had small contribution to total variation of all inflammatory markers (<10%). It could be explained by properties of zinc. Zinc has been well known as a vital micronutrient with antioxidant and anti-inflammatory properties [39] that affects various organisms [40] and thus it also has the potential to influence cardiovascular health through its interaction with cardiovascular cells [41]. The major underlying mechanism of CVD is atherosclerosis, which is promoted by inflammation [42]. Atherosclerosis is initiated with lipid oxidation by inducing the expression of vascular cell adhesion molecular-1 (VCAM-1) [42]. Of these mechanisms, pro-inflammatory cytokines (IL-1, IL-6, and TNF-α) and acute-response protein (CRP) are likely to play a role in the pathways that stimulate the expression of adhesion molecules like VCAM-1 [42,43] as well as the recruitment of monocytes [44].

We found a relatively stronger linear trend between serum zinc levels and inflammatory markers in women than in men. However, among men, IL-6 also showed the significant inverse linear trend with serum zinc level and CRP was the lowest in the highest tertile of serum zinc level, although there was no relation to TNF-α among men, unlike the inverse relation among women.

Several limitations of this study should be considered in the interpretation of our results. First, we could not draw a causal relationship between zinc status and inflammatory markers through this cross-sectional study design. Secondly, there is need to use a marker for reflecting the zinc status related to long-term dietary zinc intake at individual level. In the present study, we used serum zinc level as one marker of zinc status. Though serum zinc levels are useful in assessing zinc status at the individual level, especially in large population studies [31], measuring these levels does not appear to predict long-term dietary zinc intake. Third, study subjects were taken from only one county of South Korea and the effects of regional differences in environment, socioeconomic status, health-related habits on serum zinc level, inflammatory markers, and its relationship could not be considered. Despite these limitations, it is important to recognize that, until now, there has been no clear evidence of zinc status mechanisms playing a mediating role in inflammatory response and this is the first epidemiological study on zinc status and inflammatory markers known to affect atherosclerosis. Therefore, zinc status and inflammatory markers may provide important information regarding mechanisms of inflammatory response.

In conclusion, serum zinc levels may inversely be related to inflammatory markers (IL-6, TNF-α, and CRP), particularly in women. Although we did not observe a significant results of the dietary zinc intake relation to inflammatory markers, our results indicate that serum zinc levels could be partially responsible for lower inflammation processes in terms of acute inflammation. Further studies are needed that will investigate the precise mechanisms linking zinc, inflammation, and atherosclerosis.

## References

[1] Male D, Brostoff J, Roth D, Roitt I. Immunology: Saunders 2012.

[2] Pearson TA, Mensah GA, Alexander RW, Anderson JL, Cannon RO, Criqui M, et al Markers of inflammation and cardiovascular disease application to clinical and public health practice—A statement for healthcare professionals from the centers for disease control and prevention and the American Heart Association. Circulation 2003; 107: 499–511. 12551878 10.1161/01.cir.0000052939.59093.45

[3] Feghali CA, Wright TM. Cytokines in acute and chronic inflammation. Front Biosci 1997; 2: d12–26. 9159205 10.2741/a171

[4] Ait-Oufella H, Taleb S, Mallat Z, Tedgui A. Recent advances on the role of cytokines in atherosclerosis. Arterioscler Thromb Vasc Biol 2011; 31: 969–979. 10.1161/ATVBAHA.110.207415 21508343

[5] Kaptoge S, Seshasai SR, Gao P, Freitag DF, Butterworth AS, Borglykke A, et al Inflammatory cytokines and risk of coronary heart disease: new prospective study and updated meta-analysis. Eur Heart J 2014; 35: 578–589. 10.1093/eurheartj/eht367 24026779 PMC3938862

[6] Yousuf O, Mohanty BD, Martin SS, Joshi PH, Blaha MJ, Nasir K, et al High-sensitivity C-reactive protein and cardiovascular disease: a resolute belief or an elusive link? J Am Coll Cardiol 2013; 62: 397–408. 10.1016/j.jacc.2013.05.016 23727085

[7] Pepys MB, Baltz ML. Acute phase proteins with special reference to C-reactive protein and related proteins (pentaxins) and serum amyloid A protein. Adv Immunol 1983; 34: 141–212. 6356809 10.1016/s0065-2776(08)60379-x

[8] Ridker PM. High-sensitivity C-reactive protein potential adjunct for global risk assessment in the primary prevention of cardiovascular disease. Circulation 2001; 103: 1813–1818. 11282915 10.1161/01.cir.103.13.1813

[9] de Oliveira Otto MC, Alonso A, Lee D-H, Delclos GL, Jenny NS, Jiang R, et al Dietary micronutrient intakes are associated with markers of inflammation but not with markers of subclinical atherosclerosis. J Nutr 2011; 141: 1508–1515. 10.3945/jn.111.138115 21653577 PMC3138642

[10] De Paula R, Aneni EC, Costa APR, Figueiredo VN, Moura FA, Freitas WM, et al. Low zinc levels is associated with increased inflammatory activity but not with atherosclerosis, arteriosclerosis or endothelial dysfunction among the very elderly. BBA Clin 2014. 10.1016/j.bbacli.2014.07.002 PMC4633969 26676114

[11] Yang YJ, Choi BY, Chun B-Y, Kweon S-S, Lee Y-H, Park PS, et al Dietary zinc intake is inversely related to subclinical atherosclerosis measured by carotid intima-media thickness. Br J Nutr 2010; 104: 1202–1211. 10.1017/S0007114510001893 20487581

[12] Jung SK, Kim M-K, Lee Y-H, Shin DH, Shin M-H, Chun B-Y, et al Lower Zinc Bioavailability May Be Related to Higher Risk of Subclinical Atherosclerosis in Korean Adults. PLoS One 2013; 8. 10.1371/journal.pone.0080115 PMC3819296 24223217

[13] Liberatos P, Link BG, Kelsey JL. The measurement of social class in epidemiology. Epidemiol Rev 1988; 10: 87–121. 3066632 10.1093/oxfordjournals.epirev.a036030

[14] Ahn Y, Kwon E, Shim JE, Park MK, Joo Y, Kimm K, et al Validation and reproducibility of food frequency questionnaire for Korean genome epidemiologic study. Eur J Clin Nutr 2007; 61: 1435–1441. 17299477 10.1038/sj.ejcn.1602657

[15] The Korean Nutrition Society. Food composition table In Recommended Dietary Allowances for Koreans, 7th ed. Seoul: The Korean Nutrition Society 2011.

[16] The Korean Nutrition Society. Food Composition Table In Recommended Dietary Allowances for Koreans. Seoul: The Korean Nutrition Society 2000.

[17] Joung H, Nam G, Yoon S, Lee J, Shim E, Paik HY. Bioavailable zinc intake of Korean adults in relation to the phytate content of Korean foods. J Food Compost Anal 2004; 17: 713–724.

[18] Harland B, Oberleas D. Phytate in foods. World Rev Nutr Diet 1987; 52: 235 3327233 10.1159/000415199

[19] Tsai D-H, Amyai N, Marques-Vidal P, Wang J-L, Riediker M, Mooser V, et al Effects of particulate matter on inflammatory markers in the general adult population. Part Fibre Toxicol 2012; 9. 10.1186/1743-8977-9-24 PMC3464812 22769230

[20] Uh H-W, Hartgers FC, Yazdanbakhsh M, Houwing-Duistermaat JJ. Evaluation of regression methods when immunological measurements are constrained by detection limits. BMC Immunol 2008; 9. 10.1186/1471-2172-9-59 PMC2592244 18928527

[21] Zhao G, Etherton TD, Martin KR, Gillies PJ, West SG, Kris-Etherton PM. Dietary alpha-linolenic acid inhibits proinflammatory cytokine production by peripheral blood mononuclear cells in hypercholesterolemic subjects. Am J Clin Nutr 2007; 85: 385–391. 17284733 10.1093/ajcn/85.2.385

[22] Willett W. Nutritional epidemiology: Oxford University Press 2012.

[23] The Korean Nutrition Society. Dietary Reference Intakes for Koreans. Seoul: The Korean Nutrition Society 2010.

[24] Fabian E, Elmadfa I. Nutritional situation of the elderly in the European Union: Data of the European Nutrition and Health Report (2004). Ann Nutr Metab 2008; 52: 57–61. 10.1159/000115352 18382082

[25] U.S. Department of Agriculture ARS (2014) Nutrient Intakes from Food and Beverages: Mean Amounts Consumed per Individual, by Gender and Age, *What We Eat in America*, NHANES 2011–2012.

[26] de Benoist B, Darnton-Hill I, Davidsson L, Fontaine O, Hotz C. Conclusions of the joint WHO/UNICEF/IAEA/lZiNCG Interagency meeting on zinc status indicators. Food Nutr Bull 2007; 28: S480–S484. 17988008 10.1177/15648265070283S306

[27] Lowe NM, Fekete K, Decsi T. Methods of assessment of zinc status in humans: a systematic review. Am J Clin Nutr 2009; 89: 2040S–2051S. 10.3945/ajcn.2009.27230G 19420098

[28] Hess SY, Peerson JA, King JC, Brown KH. Use of serum zinc concentration as an indicator of population zinc status. Food Nutr Bull 2007; 28: S403–S429. 17988005 10.1177/15648265070283S303

[29] Roohani N, Hurrell R, Kelishadi R, Schulin R. Zinc and its importance for human health: An integrative review. J Res Med Sci 2013; 18: 144–157. 23914218 PMC3724376

[30] Andriollo-Sanchez M, Hininger-Favier I, Meunier N, Toti E, Zaccaria M, Brandolini-Bunlon M, et al Zinc intake and status in middle-aged and older European subjects: the ZENITH study. Eur J Clin Nutr 2005; 59: S37–S41. 16254579 10.1038/sj.ejcn.1602296

[31] Wood RJ. Assessment of marginal zinc status in humans. J Nutr 2000; 130: 1350S–1354S. 10801942 10.1093/jn/130.5.1350S

[32] Seo JA, Song SW, Han K, Lee KJ, Kim HN. The associations between serum zinc levels and metabolic syndrome in the Korean population: findings from the 2010 Korean National Health and Nutrition Examination Survey. PLoS One 2014; 9: e105990 10.1371/journal.pone.0105990 25153887 PMC4143320

[33] Kogirima M, Kurasawa R, Kubori S, Sarukura N, Nakamori M, Okada S, et al Ratio of low serum zinc levels in elderly Japanese people living in the central part of Japan. Eur J Clin Nutr 2007; 61: 375–381. 16969379 10.1038/sj.ejcn.1602520

[34] Gibson RS, Heath A-LM, Limbaga MLS, Prosser N, Skeaff CM. Are changes in food consumption patterns associated with lower biochemical zinc status among women from Dunedin, New Zealand? Br J Nutr 2001; 86: 71–80. 11432767 10.1079/bjn2001370

[35] Lowe NM, Medina MW, Stammers A-L, Patel S, Souverein OW, Dullemeijer C, et al The relationship between zinc intake and serum/plasma zinc concentration in adults: a systematic review and dose-response meta-analysis by the EURRECA Network. Br J Nutr 2012; 108: 1962–1971. 10.1017/S0007114512004382 23244547

[36] Reeves PG. Adaptation responses in rats to long-term feeding of high-zinc diets: emphasis on intestinal metallothionein. J Nutr Biochem 1995; 6: 48–54.

[37] de Oliveira Otto MC, Alonso A, Lee D-H, Delclos GL, Bertoni AG, Jiang R, et al Dietary intakes of zinc and heme iron from red meat, but not from other sources, are associated with greater risk of metabolic syndrome and cardiovascular disease. J Nutr 2012; 142: 526–533. 10.3945/jn.111.149781 22259193 PMC3278268

[38] Lee DH, Folsom AR, Jacobs DR. Iron, zinc, and alcohol consumption and mortality from cardiovascular diseases: the Iowa Women's Health Study. Am J Clin Nutr 2005; 81: 787–791. 15817853 10.1093/ajcn/81.4.787

[39] Prasad AS. Clinical, immunological, anti-inflammatory and antioxidant roles of zinc. Exp Gerontol 2008; 43: 370–377. 18054190 10.1016/j.exger.2007.10.013

[40] Maret W. Zinc and human disease. Met Ions Life Sci 2013; 13: 389–414. 10.1007/978-94-007-7500-8_12 24470098

[41] Little PJ, Bhattacharya R, Moreyra AE, Korichneva IL. Zinc and cardiovascular disease. Nutrition 2010; 26: 1050–1057. 10.1016/j.nut.2010.03.007 20950764

[42] Libby P. Inflammation and cardiovascular disease mechanisms. Am J Clin Nutr 2006; 83: 456s–460s. 16470012 10.1093/ajcn/83.2.456S

[43] Pasceri V, Willerson JT, Yeh ET. Direct proinflammatory effect of C-reactive protein on human endothelial cells. Circulation 2000; 102: 2165–2168. 11056086 10.1161/01.cir.102.18.2165

[44] Torzewski M, Rist C, Mortensen RF, Zwaka TP, Bienek M, Waltenberger J, et al C-reactive protein in the arterial intima: role of C-reactive protein receptor-dependent monocyte recruitment in atherogenesis. Arterioscler Thromb Vasc Biol 2000; 20: 2094–2099. 10978254 10.1161/01.atv.20.9.2094

